# Three-Dimensional Posture Estimation of Vehicle Occupants Using Depth and Infrared Images

**DOI:** 10.3390/s24175530

**Published:** 2024-08-27

**Authors:** Anuj Tambwekar, Byoung-Keon D. Park, Arpan Kusari, Wenbo Sun

**Affiliations:** 1 Department of Computer Science and Engineering, University of Michigan, Ann Arbor, MI 48109, USA; anujt@umich.edu; 2 University of Michigan Transportation Research Institute, Ann Arbor, MI 48109, USA; keonpark@umich.edu (B.-K.D.P.); kusari@umich.edu (A.K.)

**Keywords:** posture estimation, computer vision, depth-sensing, LiDAR, vehicular occupant safety

## Abstract

Pose estimation is crucial for ensuring passenger safety and better user experiences in semi- and fully autonomous vehicles. Traditional methods relying on pose estimation from regular color images face significant challenges due to a lack of three-dimensional (3D) information and the sensitivity to occlusion and lighting conditions. Depth images, which are invariant to lighting issues and provide 3D information about the scene, offer a promising alternative. However, there is a lack of strong work in 3D pose estimation from such images due to the time-consuming process of annotating depth images with 3D postures. In this paper, we present a novel approach to 3D human posture estimation using depth and infrared (IR) images. Our method leverages a three-stage fine-tuning process involving simulation data, approximated data, and a limited set of manually annotated samples. This approach allows us to effectively train a model capable of accurate 3D pose estimation with a median error of under 10 cm across all joints, using fewer than 100 manually annotated samples. To the best of our knowledge, this is the first work focusing on vehicle occupant posture detection utilizing only depth and IR data. Our results demonstrate the feasibility and efficacy of this approach, paving the way for enhanced passenger safety in autonomous vehicle systems.

## 1. Introduction

With the rise in popularity of semi-autonomous vehicles and ongoing research towards fully autonomous vehicles progressing rapidly, drivers and passengers are now able to do a lot more than merely sit or drive in a car. Depending on the level of automation of the vehicle, occupants may have different postures [1]. Traditionally, crash tests and safety mechanisms have been designed for general seating, with very few “out-of-position postures” that are considered. Prior research indicates that with the increase in automation, there is a need to adapt to occupants’ postures in order to improve their safety [2]. Thus, the safety mechanisms and tests for proving safety would need to evolve for these non-standard postures. But, in order to do that, firstly, an accurate and robust estimation of occupants’ postures is vital.

While accurate marker-based posture capture mechanisms exist [3], they require multiple cameras inside the vehicle, and markers placed on the occupants, making them impractical for practical deployment. Markerless posture capture mechanisms have shown remarkable promise but mainly operate on RGB images and are insufficiently accurate, either estimating 2D postures instead of 3D or estimating 3D postures from 2D data. In addition to these challenges, using color images of occupants of a vehicle may lead to privacy preservation issues, as occupants would need to be comfortable with cameras tracking their activity. LiDAR data provide an interesting avenue to mitigate some of these problems, as it is possible to obtain highly accurate depth information, thereby obtaining 3D data instead of just 2D data while preserving occupant privacy. Unfortunately, LiDAR data can often be hard to work with when performing keypoint estimation tasks owing to the lack of fine-grained detail that can be used to pick out key human-related features in crowded scenes. Infrared (IR) images can be used to pick out people from crowded scenes and are substantially more fine-grained than depth images but do not provide the extensive depth information that LiDAR images provide.

In this work, we leverage the advantages of LiDAR and IR images to develop a deep learning-based model that predicts occupant posture using solely LiDAR and infrared (IR) images. Using just the LiDAR and IR images, we are able to accurately predict the location of the key joints of the occupant with respect to the body center. In order to achieve this, we use a three-step fine-tuning process, where we sequentially train a model on three datasets—simulation data, domain adaptation data, and manually annotated data. This three-step process allows us to successfully train a deep model without the need to manually annotate a large amount of data.

The key contributions of this work are:

- A novel training approach to train accurate posture estimation neural networks using solely IR and depth images;
- A method to reduce the amount of manually annotated samples required to train posture estimation networks, thereby reducing the costs and effort required to create posture-estimation datasets.

## 2. Related Work

Keypoint and posture estimation can be classified on the nature of the output (2D vs. 3D) and the type of input (color, RBGD, and exclusively depth). Two-dimensional keypoint detection and posture estimation techniques predict the location of joints or keypoints in two dimensions, locating the joints on the 2D input. YOLO [4] and OpenPose [5] are two of the most popular and best-performing 2D pose estimation methods, both of which use deep learning. YOLO was originally conceived as a proposal-free object detection pipeline and was later fine-tuned on MS-COCO [6] to perform 2D keypoint estimation. While fast and accurate in most situations dealing with color or greyscale images, YOLO exhibits poor performance when used with depth images, owing to the lack of any distinct facial structure in the images. OpenPose uses a more tailored approach, learning part-affinity fields that allow the model to correlate sections of images with various body parts. OpenPose’s key benefit over YOLO is the ability to predict 3D postures when multiple viewpoints are provided to the model. While powerful, OpenPose’s 3D posture estimation is still reliant on RBG images as input, and, as seen later in Section 4.2, we find that it is still not very accurate when it comes to posture prediction of vehicular occupants when used with depth images. Ref. [7] showcases multiple other 3D pose estimation techniques from 2D inputs, but all the highlighted techniques operate on color images, with no prior work focusing on pose estimation from LiDAR or depth images. Ref. [8] focuses on 3D model reconstruction from LiDAR data, but uses 3D scans of subjects to create point clouds, which are then refined into subject-specific models. Additionally, they focus on generating the base body mesh of the subject as opposed to predicting the actual posture that the subject is exhibiting. Ref. [9] uses single frame depth images, which is the closest to our input, but focuses exclusively on predicting hand poses instead of full-body posture. Ref. [10] focuses on a similar task of vehicle occupant posture prediction but uses RGB and near-infrared (NIR) images instead of LiDAR.

This gap in the existing research using depth images for 3D posture prediction is likely due to the lack of any extensive datasets focused on this task. While datasets like Human3.6M [11] have 3D estimates for natural 2D poses, no datasets exist for joint location estimation from LiDAR and IR data, nor are there any datasets focused on 3D posture estimation specifically for vehicle occupants whose postures are substantially different from the kind seen in Human3.6M and MS-COCO. HIVE [10] contains RGB and IR image data for vehicle occupants but lacks any 3D or LiDAR data.

Our work is motivated by these gaps in techniques and data, and our three-step fine-tuning process leverages recent advances in statistical human body-shape modeling to help mitigate the challenges associated with creating large annotated posture datasets for LiDAR data. To the best of our knowledge, this work is the first study using LiDAR and IR data for posture estimation. Additionally, we provide not only an architecture to estimate postures via LiDAR and IR data but also describe a general dataset generation and training methodology that enables posture-estimation networks to be trained with a very small number of annotated samples.

## 3. Methodology

### 3.1. Problem Formulation

We start with notations and problem formulations to formally describe the target problem. The objective is to identify the posture of subjects using depth and IR images. A posture is defined as 3D locations of *m* key joints in a human body, using the body center as the origin. *m* is set as 15 in the study, which omits the joints on lower extremities obscured by interior components of vehicles. These joints are the pelvis, abdomen, thorax, neck, head, left hip, left knee, right hip, right knee, left shoulder, left elbow, left wrist, right shoulder, right elbow and right wrist. We denote scalars or vectors by using lowercase letters and denote matrices or tensors by using uppercase letters throughout the paper. Let *Z* denote the posture as a matrix of m×3. Let *s* denote the subject covariates describing the subject size. The subject body mesh is a function of *Z* and *s*, represented by ψ(Z,s) [12].

In-vehicle cameras are employed to measure the body mesh as depth images and infrared (IR) images. Let *c* denote camera parameters, including the camera location and angle. The depth and IR images *Y* depend on the body mesh and camera parameters and hence can be written as

(1) Y=η(ψ(Z,s),c),

where η is the unknown projection function. To capture postures with precision, two cameras are installed to collect depth and IR images, characterized by the parameters $c_{1}$ and $c_{2}$. The objective of this study is to develop a function that estimates the posture *Z* based on the measurements and camera parameters collected in $\left(Y,s,c_{1},c_{2}\right)$. Our approach is learning-based, that is, to train a neural network $f_{\theta }$ such that $\hat{Z}=f_{\theta }\left(Y,s,c_{1},c_{2}\right)$ can well approximate *Z*. The neural network parameter θ is estimated through solving the optimization problem:

(2) $$\hat{\theta }=\underset{\theta }{\mathrm{argmin}}\;\sum_{\left(Y,s,c_{1},c_{2},Z\right)\in D}∥Z-f_{\theta }\left(Y,s,c_{1},c_{2}\right)∥,$$

where ∥·∥ represents the L2 loss that measures the discrepancy between the predicted and true joint locations in the Euclidean space and D denotes the training dataset. In what follows, we will elaborate the construction of the training dataset D and the neural network architecture $f_{\theta }$.

### 3.2. Data Collection

Following the protocol in [13], depth and IR images were collected from two Microsoft Kinect V2s on the dashboard of a car, while vehicle occupants were asked to perform various tasks. This dataset contains a large number of highly detailed depth and IR images for a host of various occupant postures. However, the dataset does not contain ground truth postures *Z*, making the direct neural network training infeasible. While it may seem trivial to annotate this data, it is a time-consuming and difficult process, with a single sample requiring five to ten minutes to annotate.

To overcome this challenge, a fine-tuning-based domain-aware posture method is developed. The basic concept is to estimate θ in a data-rich environment using simulation data and then update θ to account for real-world variation via fine-tuning on limited manually annotated data. The proposed approach requires us to extend the training dataset D to involve three components—simulation data ($D_{s}$), automatically annotated data ($D_{o}$), and manually annotated data ($D_{m}$).

**Figure 1:**
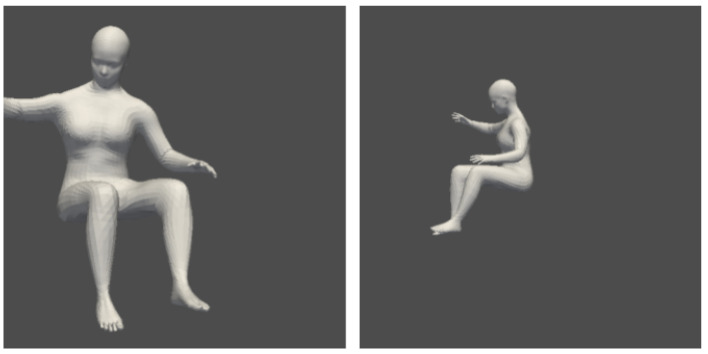
Left and right camera views for a simulation data point.

In particular, using the Statistical Body Shape Model [12], we synthetically generate a large dataset of simulated depth images corresponding to various postures, denoted by $D_{s}$. This is performed by creating 50,000 joint angle vectors by performing Latin hypercube sampling of possible joint angles for each of the 15 joints of interest. These joint angle vectors are then paired with the anthropometric data of 20 subjects at random to create 50,000 unique body meshes, each of whose joint locations *Z* is exactly known. We then convert each mesh into two depth images by creating two cameras with randomized initial locations and obtaining the corresponding depth projection. We keep 40,000 of these data points for model training, and the remaining 10,000 for validation. It is important to note that these depth images are solely of body shape models (effectively, just mannequins) and contain no trace of the vehicle, its interior, or items of clothing that can be on a person that would change the depth image perceived by the sensor. Figure 1 provides an example of a simulated example where the left and right cameras capture two different profiles of the mesh.

While $D_{s}$ contains exact joint location values, it fails to account for measurement noise and human variation that would be expected during data collection in the real-world setting. The depth images in $D_{s}$ only contain the body meshes, while the actual data in [13] are noisy and are situated inside a vehicle, resulting in a completely different background when observing the IR and depth images. In addition, the subjects in the real data are wearing clothing, which can fold and crease creating a much more varied depth image. There are also numerous foreign objects in the images in the real-world data, such as water bottles in a side compartment, a laptop on the subject’s lap, or even glasses on their face. This variation in the simulation and real data makes domain awareness a necessity when adapting a model from simulation data to real-world data.

Instead of manually labeling the posture *Z* throughout the real dataset, we apply OpenPose [5] to IR images to achieve approximations of *Z*. In particular, 2D locations of key joints in IR images are obtained through OpenPose. The depth value at the 2D joint locations is collected from the depth image to form the complete 3D information for *Z*. Note that the depth value measures the distance from the camera to the surface of the body mesh instead of the actual joint. We hence use two cameras to accurately estimate the joint location by finding the intersection between the lines connecting an estimated surface-level joint location to the camera that captured it. This procedure is applied to 3000 IR–depth image pairs, resulting in 2500 training samples and 500 validation samples. The dataset is denoted by $D_{o}$.

OpenPose joint estimates are not always accurate, and the estimated values in $D_{o}$ are not sufficiently accurate to enable dynamic adaptive safety devices. In order to improve performance, we create $D_{m}$ by manually adjusting and fitting body meshes to the real depth images. Due to the labor cost of the manual annotation procedure, 96 samples are labeled from 16 postures of 6 subjects, with 81 used for training and 15 for validation. To this end, the manually annotated dataset is denoted by $D_{m}$. A summary of the dataset structure is provided in Table 1. The data for the six selected subjects can be found in Table 2.

### 3.3. Domain-Aware Posture Estimation

Data processing is conducted for the datasets $D_{o}$ and $D_{m}$. In the two datasets, postures are normalized such that occupants are centered and facing the same direction. The pelvis is used as the body center with its position set as the origin of the 3D coordinates. To ensure consistency during training, we rotate the target posture such that the left hip is along the −y axis, and the abdomen is along +z axis. This rotation ensures that all postures are facing the same direction, without introducing any assumptions about the posture itself.

**Figure 2:**
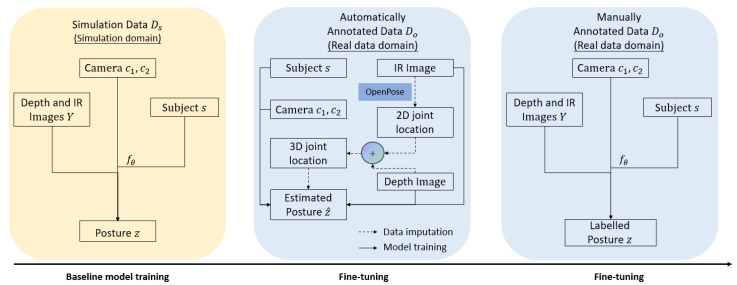
Flowchart of the three-stage training process.

Given the three datasets with distinct features, a three-step learning scheme is proposed for domain-aware posture estimation. A baseline model is firstly trained on the simulation data $D_{s}$ with complete information for $\left(Y,s,c_{1},c_{2},Z\right)$. The baseline model is then fine-tuned with the automatically annotated data $D_{o}$ whose *Z* is estimated via utilizing the pre-trained OpenPose model. Finally, the manually labeled data $D_{m}$ are introduced to further fine-tune the model to obtain a close-to-reality posture estimation model. The method framework is illustrated in Figure 2.

**Figure 3:**
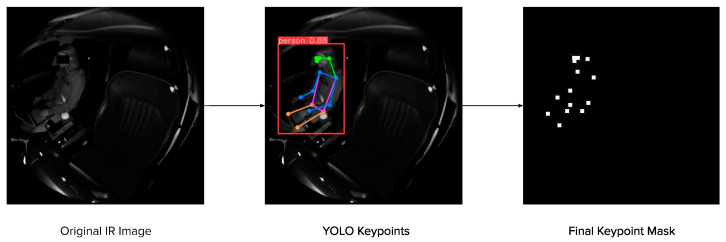
Input processing: The IR image from each camera is first scaled and then passed through YOLOv8 to obtain estimated 2D keypoints. These 2D keypoints are then processed to create a keypoint mask. (Note: The black box obscuring the face in the original IR image has been added to preserve subject privacy.)

**Figure 4:**
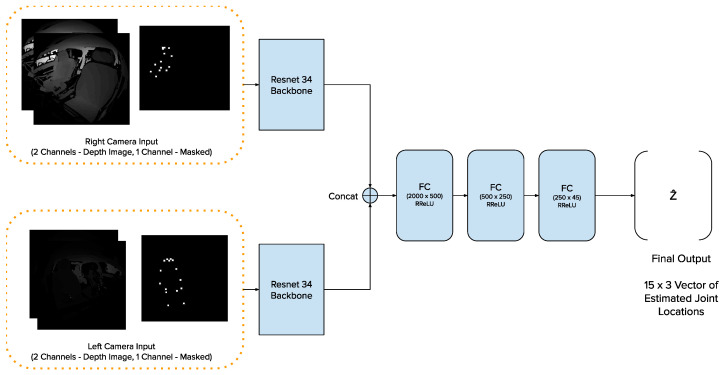
Model architecture $f_{\theta }$: The depth images and masked depth image are stacked to create two 3-channel inputs, one per camera. These inputs are passed to the two ResNet blocks of the model, which ultimately produces 45 output values from the last fully connected layer. These outputs are reshaped to a 15×3 vector to create the final output, which contains 3 coordinate values for each of the 15 joints. In the visualization shown, the depth images have been converted to greyscale—pixels closer to the camera are darker, while those further away are lighter. The backbone architecture can be seen in detail in Figure 5.

**Figure 5:**
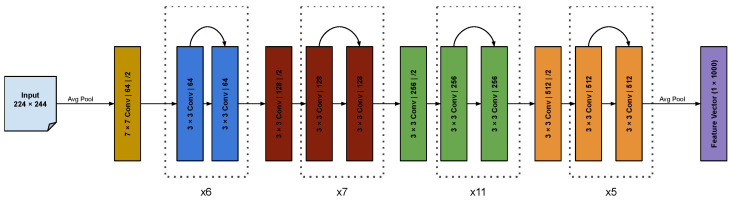
Architecture of the Resnet 34 backbone used in the posture estimation model.

The model architecture for $f_{\theta }$ is designed as follows. We use two ResNet34 [14] models as image backbones and pass them through three fully connected layers with the ReLU activation function [15], with a final output size of 45 (3 coordinates for each of the 15 joint locations). The ResNet architecture was chosen as the backbone due to its success in object detection, semantic segmentation, and keypoint detection networks [16,17]. Resnet34 was chosen since we have two backbones (one per viewpoint) instead of one, resulting in more parameters than the typical ResNet50 model used in these networks. Resnet50 would result in over a 100 total backbone layers, which is both computationally very demanding to train and would also make the model at risk for overparameterization and overfitting. To aid with noise reduction while retaining prominent features necessary for estimating joint locations, we first obtain 2D joint locations on the IR images using YOLO [4] and create masks that are overlaid on the depth image (see Figure 3). These filtered depth images are combined with the original depth images to create the input that is passed through our network to obtain a set of estimated joint locations (Figure 4). The architecture used in the backbones can be seen in Figure 5.

The layers after the two ResNet blocks use the randomized leaky ReLU (RReLU) activation function due to its reported better stability and better performance on standard image-related tasks [18]. We note that the selection of a ReLU-variant activation appears to be very important for this task, as initial experiments with non-ReLU activations for any layer resulted in very poor performance. Figure 2 provides an illustration of how the three-step training process is performed.

### 3.4. Model Training Parameters and Hardware

The base model was trained on simulation data for 100 epochs using the Adam optimizer [19] with a learning rate of 0.001. The model with the highest validation performance on the simulation validation set was used for the further steps. For both subsequent steps, the model was trained for 50 epochs with a learning rate of 0.0001, this time using the AdamW optimizer [20], with a weight decay of $5\times 10^{-4}$ and AMSGrad [21] enabled. All model development and training was performed on Python 3.8, with Pytorch version 2.1.0. Training was performed on the University of Michigan’s Great Lakes computing server, on nodes with 4 CPU cores, 16 GB of RAM, and a single NVIDIA Tesla V100 GPU, running the Unix Red Hat operating system.

## 4. Results

### 4.1. Results on Simulation Data

**Figure 6:**
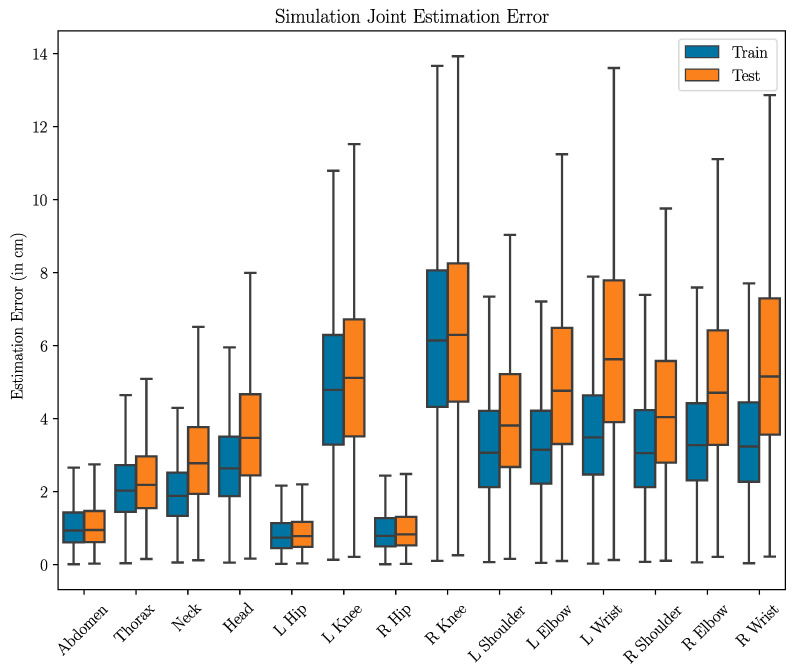
Boxplot of per-joint estimation error for the simulation data. Blue boxes represent the training data, while orange boxes represent the testing data.

To validate our model architecture and confirm that we could indeed estimate joint locations from depth data, we first trained the neural network on the simulation dataset only. Our results indicate that the model architecture can indeed learn and estimate joint locations accurately on the simulation dataset, with per-joint average errors on the test data being under 6.5 cm. The results also indicate that, as expected, joints further from the body center are harder to estimate, particularly the hands, since the allowable range of motion and positions is larger. Figure 6 provides a visualization of the uncertainty of the per-joint estimations (average errors are provided in the Appendix A in Table A1).

### 4.2. Results on Real Data

While the errors on the simulation data via the baseline model are low, training on only simulation data is insufficient. Ref. [22] shows how convolutional networks can fail when moving from one autonomous driving dataset to another. In a similar fashion, our real data consist of noisy LiDAR data where parts of the passengers’ bodies are occluded in the 3D scan. This results in incomplete meshes which can contain holes. Additionally, the position of the occupants, their clothing, and the items they bring into the vehicle like their laptops or water bottles all result in variations within the real depth images that simply do not exist in the simulation. Attempting to use the model trained on only simulator data with the real data results in catastrophic failure—the model simply predicts the average posture each time. To fix this, we further fine-tune the model with OpenPose and manually fitted data as outlined in Section 3.3.

**Figure 7:**
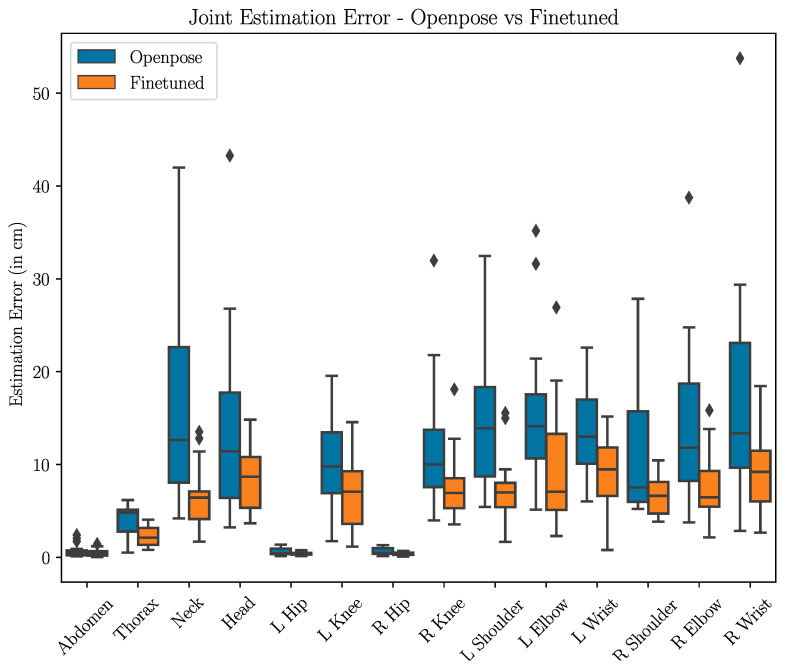
Boxplot of per-joint estimation errors of the OpenPose-trained model (blue, on the left) versus the fine-tuned model (orange, on the right). The fine-tuned model results in significantly lower average and median error, with a substantially reduced error distribution. Errors in the abdomen and hip joints are minimal owing to the normalization process and the rigidity of the pelvis.

Figure 7 showcases the results of the OpenPose-trained model (i.e., training the model with steps 1 and 2 in Figure 2) and the fine-tuned model (i.e, training the model with all steps in Figure 2). We find that the fine-tuned model significantly outperforms the OpenPose-trained model for all joints in terms of average estimation error, with the fine-tuned model achieving roughly 10 cm or less of average error (average errors are provided in the Appendix A in Table A2). As seen in the figure, fine-tuning results in a substantial reduction in the median error of all joints, with all joints in the fine-tuned model having a median error of under 10 cm. The fine-tuned model also exhibits a nearly 50% reduction in maximum error across the board. The boxplot also reveals that the highest average error joint—the left shoulder—is due to the presence of outliers with large estimation errors. The fine-tuning process results in a significant reduction in not only the magnitude of the error but also the degree of variation in error, which is of critical importance in safety applications for vehicle occupants.

**Figure 8:**
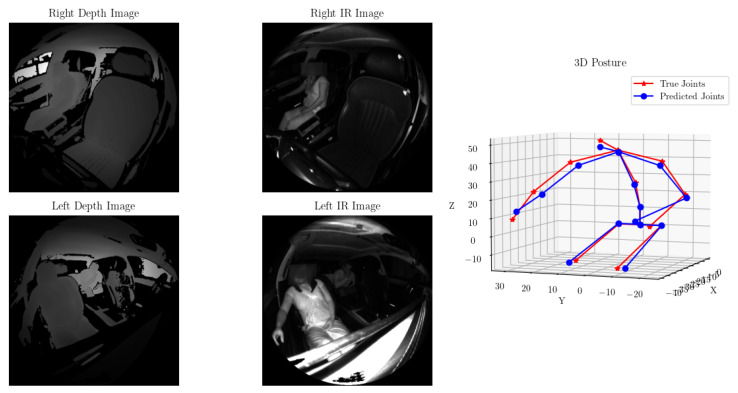
Predicted (blue) vs. actual (red) joint locations for one test sample. The predicted posture accurately captures the outstretched right arm and lean of the occupant.

Manual verification of the predicted joint locations vs. the ground truth locations revealed that in nearly all cases, the predicted posture was well aligned with the true postures. Figure 8 showcases one such example.

**Figure 9:**
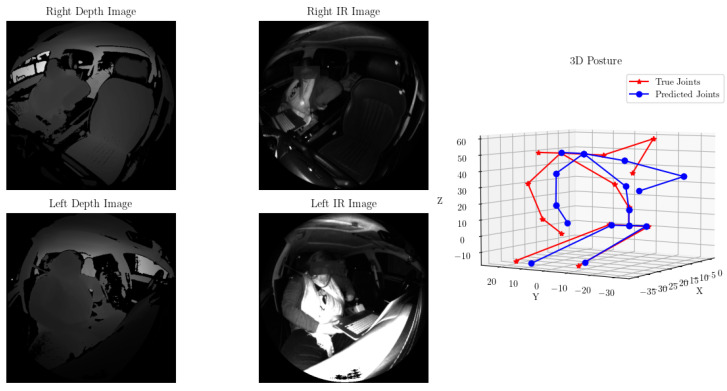
The poorest-performing test sample. Note the posture of the left arm is out of sync, primarily due to the incorrect prediction in the left elbow.

As mentioned earlier, occluded and contorted body shapes are the most challenging to predict. Figure 9 highlights the sample with the largest error. We hypothesize that this is likely due to the left arm’s depth and IR information not being visible in the left camera’s data, resulting in only one camera’s data being used to estimate the joint, combined with the additional contortion of the body resulting in a more out-of-distribution sample. Note that in all figures containing passenger IR data, the dark stripe across the face in the IR images was added afterward to obscure the faces and preserve the privacy of the participants in the study. These artifacts are not present in the actual data.

To summarize, we find that our technique estimates joint positions accurately and consistently within 10 cm of expert-annotated joint locations. Most importantly, our technique allows a sharp reduction in annotation time. Manual annotation of joint locations took us upwards of 5 min per sample as we had to manually fit the statistical body-shape model to a point cloud. On the contrary, our pipeline can predict the joint location for a single sample within 30 ms, with the input pre-processing taking 2.9 ms on average, and the model predictions taking 24.8 ms on average when using an NVIDIA Tesla V100 GPU (Santa Clara, CA, USA). As such, our technique shows a promising avenue for use cases where margins of error within 10 cm are acceptable and prediction time is of utmost importance. Downstream use cases such as occupant behavior prediction and distracted driving detection could benefit from this technique.

## 5. Conclusions and Scope for Extension

In conclusion, we find that deep convolution models can indeed estimate joint locations of vehicle occupants using depth images, as long as they are appropriately trained. Traditional off-the-shelf posture detection models are reliant on distinct facial features that are absent in depth images, making it necessary to train models on labeled depth data from scratch. The proposed three-stage fine-tuning process allows us to leverage advancements in 3D human modeling and 2D pose estimation to circumvent the challenges associated with annotating depth data with detailed posture information.

This work encourages the creation of dedicated LiDAR and depth-image datasets catered to human posture estimation. While our results are promising, we acknowledge that our manual annotation and test sample size is very limited. A model trained on a large number of expert annotations is surely likely to perform better than our technique, and so we hope that this work encourages additional research into the development of large-scale human posture datasets that use exclusively LiDAR data.

In addition to this, our model has some drawbacks and caveats. For one, our testing was conducted on data from adults in the front passenger seat. As such, this model will likely fail to accurately predict joints for an occupant in a different seat or a child. Additionally, our approach assumes that the camera feed contains a single occupant, and as such, may produce incorrect results when multiple subjects are present in the scene. We hope to address these limitations in future work to create a stronger and more robust posture determination system for downstream tasks. 

## Figures and Tables

**Table 1:** The three components of the dataset D.

Component	Type of Input *Y*	*Z*	Number of Data Points	Function
$D_{s}$	Simulated	Exact	50,000	Pre-training
$D_{o}$	Real	Approximate	3000	Fine-tuning
$D_{m}$	Real	Exact	81	Fine-tuning

**Table 2:** Data for subjects used for manual annotation.

ID	Sex	Age	Stature	BMI	Sitting Height	Waist Circ.
1	F	21	1590	25.157	855	745
2	F	36	1530	25.610	836	832
3	F	30	1650	23.380	884	826
4	M	20	1698	22.580	909	775
5	M	28	1853	25.630	983	878
6	F	28	1607	38.684	910	786

**Figure A1:**
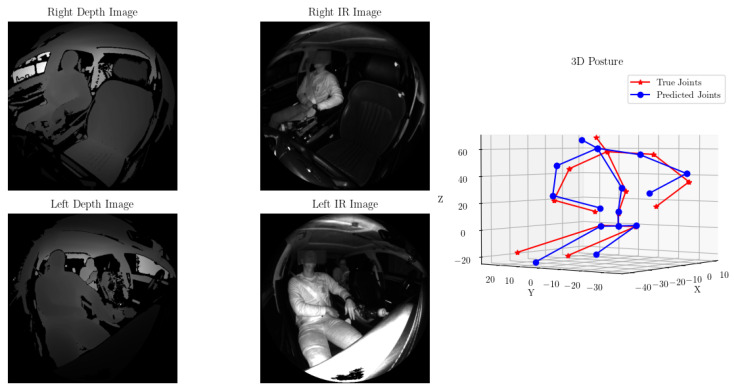
Another example of a well-estimated posture, where the predicted posture accurately captures the twist in the spine, as well as the arm positions, with some minor error in the Z estimate of the knees.

**Figure A2:**
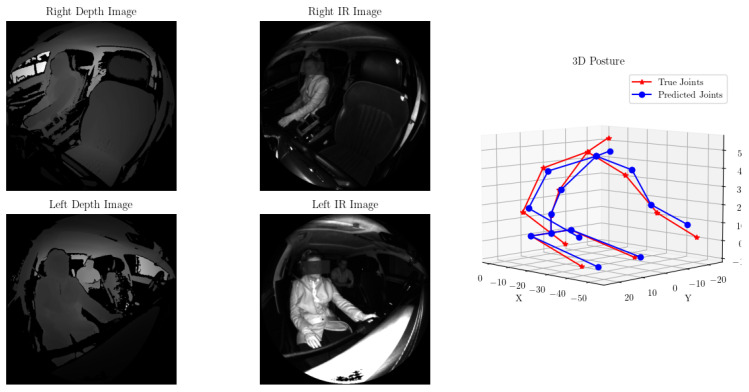
Similar to Figure 8, the estimated posture is in sync with the true predictions, accurately capturing the extended arm interacting with the console.

**Figure A3:**
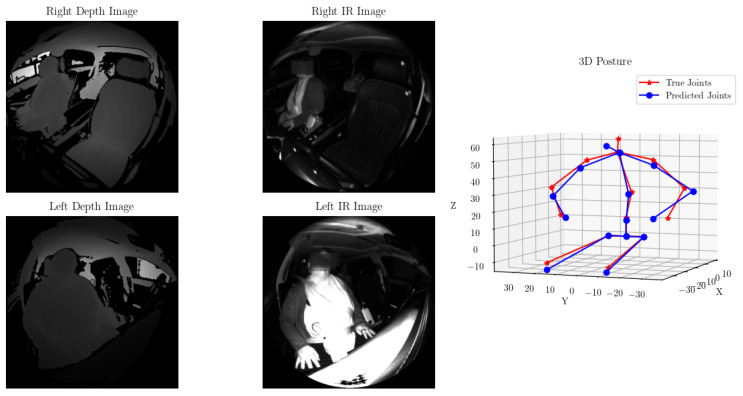
Prediction for a very standard “resting posture” of a subject. The model is able to estimate the posture with very little error. Note that the joint locations are not on the surface of the subject but inside their body meshes, which is why the joint locations of the arms may appear higher than expected on initial inspection.

## Data Availability

Data are unavailable due to privacy restrictions.

## Appendix A

### Appendix A.1. Per-Joint Average Error for Simulation Data

Table A1 provides the per-joint average error for the simulation data results described in Section 4.1.

**Table A1:** Per-Joint Average Error (in cm) for Simulation data. These results indicate that the model is sufficient to learn the task, with limb extremities being the most difficult joints to estimate.

Joint	Average Train Error (cm)	Average Test Error (cm)
Pelvis	0.84	0.88
Abdomen	1.15	1.19
Thorax	2.14	2.31
Neck	1.98	2.97
Head	2.78	3.72
Left Hip	0.82	0.86
Left Knee	4.85	5.23
Right Hip	0.92	0.96
Right Knee	6.29	6.41
Left Shoulder	3.30	4.10
Left Elbow	3.33	5.24
Left Wrist	3.68	6.38
Right Shoulder	3.32	4.37
Right Elbow	3.48	5.14
Right Wrist	3.50	5.87

### Appendix A.2. Per-Joint Average Error on Real Data

Table A2 provides the per-joint average error for the simulation data results described in Section 4.2.

**Table A2:** Per-joint average error (in cm) for occupants in the front passenger seat. The OpenPose-trained model is trained on only estimated postures using OpenPose, while the fine-tuned model is first trained with postures estimated using OpenPose and then further fine-tuned with manually annotated samples.

Joint	OpenPose-Trained Model	Fine-Tuned Model
Pelvis	0.06	0.04
Abdomen	0.90	0.76
Thorax	4.21	2.37
Neck	16.60	6.17
Head	15.62	7.37
Left Hip	0.82	0.38
Left Knee	11.41	7.99
Right Hip	0.79	0.42
Right Knee	12.93	8.89
Left Shoulder	15.10	7.05
Left Elbow	15.65	10.14
Left Wrist	13.59	8.85
Right Shoulder	11.82	6.84
Right Elbow	14.77	7.65
Right Wrist	15.98	9.19

### Appendix A.3. Additional Examples of Predictions on Real Data

Figure A1, Figure A2 and Figure A3 showcase additional examples of how our technique is able to accurately estimate joint locations.
